# Src Kinase Dependent Rapid Non-genomic Modulation of Hippocampal Spinogenesis Induced by Androgen and Estrogen

**DOI:** 10.3389/fnins.2018.00282

**Published:** 2018-05-01

**Authors:** Mika Soma, Jonghyuk Kim, Asami Kato, Suguru Kawato

**Affiliations:** ^1^Department of Cognitive Neuroscience, Faculty of Pharma-Science, Teikyo University, Itabashi, Japan; ^2^Department of Urology, Graduate School of Medicine, Juntendo University, Hongo, Japan; ^3^Department of Biophysics and Life Sciences, Graduate School of Arts and Sciences, The University of Tokyo, Meguro, Japan

**Keywords:** androgen, estrogen, Src kinase, spine, hippocampus, non-genomic, synapse

## Abstract

Dendritic spine is a small membranous protrusion from a neuron's dendrite that typically receives input from an axon terminal at the synapse. Memories are stored in synapses which consist of spines and presynapses. Rapid modulations of dendritic spines induced by hippocampal sex steroids, including dihydrotestosterone (DHT), testosterone (T), and estradiol (E2), are essential for synaptic plasticity. Molecular mechanisms underlying the rapid non-genomic modulation through synaptic receptors of androgen (AR) and estrogen (ER) as well as its downstream kinase signaling, however, have not been well understood. We investigated the possible involvement of Src tyrosine kinase in rapid changes of dendritic spines in response to androgen and estrogen, including DHT, T, and E2, using hippocampal slices from adult male rats. We found that the treatments with DHT (10 nM), T (10 nM), and E2 (1 nM) increased the total density of spines by ~1.22 to 1.26-fold within 2 h using super resolution confocal imaging of Lucifer Yellow-injected CA1 pyramidal neurons. We examined also morphological changes of spines in order to clarify differences between three sex steroids. From spine head diameter analysis, DHT increased middle- and large-head spines, whereas T increased small- and middle-head spines, and E2 increased small-head spines. Upon application of Src tyrosine kinase inhibitor, the spine increases induced through DHT, T, and E2 treatments were completely blocked. These results imply that Src kinase is essentially involved in sex steroid-induced non-genomic modulation of the spine density and morphology. These results also suggest that rapid effects of exogenously applied androgen and estrogen can occur in steroid-depleted conditions, including “acute” hippocampal slices and the hippocampus of gonadectomized animals.

## Introduction

Accumulating evidence over the past two decades supports the conclusion that some sex steroid responses in the brain involve rapid non-genomic mechanisms (Mukai et al., 2010) in addition to slow/genomic actions (Gould et al., 1990; Woolley et al., 1990; Woolley and McEwen, 1992; MacLusky et al., 2005). In rat and mouse hippocampus, androgen, and estrogen, including testosterone (T), dihydrotestosterone (DHT), and estradiol (E2), induced rapid modulation of dendritic spines, which occurred between 30 and 120 min after the application (MacLusky et al., 2005; Murakami et al., 2006; Mukai et al., 2007). Sex steroid-induced rapid effects were also observed in electrophysiological investigations, such as the long-term potentiation (LTP) (Foy et al., 1999; Bi et al., 2000; Grassi et al., 2011; Ooishi et al., 2012b; Hasegawa et al., 2015). E2-induced rapid synaptic modulation occurred not only *in vitro* but also *in vivo* conditions in the hippocampus (MacLusky et al., 2005) (Luine and Frankfurt, 2012; Luine, 2014). The rapid signaling of E2 may depend on multiple kinases, including mitogen-activated protein kinase (MAPK) and phosphoinositide 3-kinase (PI3K) in the hippocampus (Bi et al., 2000, 2001; Znamensky et al., 2003; Mannella and Brinton, 2006; Mukai et al., 2010; Luine, 2014; Tuscher et al., 2016). The contribution of many other essential serine/threonine kinases have also been studied. These important kinases are LIM domain kinase (LIMK), protein kinase A (PKA), and protein kinase C (PKC) all of which are essential for regulation of the synaptic plasticity (Frick, 2015; Hasegawa et al., 2015; Tuscher et al., 2016).

On the other hand, androgen (T and DHT)-induced rapid effects on synaptic modulation in the hippocampus or other brain regions have not been extensively investigated (Foradori et al., 2008; Hajszan et al., 2008), while rapid effects of androgen were extensively studied in prostate cancer cells and gonadal cells (Migliaccio et al., 2000; Cheng et al., 2007). We tried to examine molecular mechanisms of rapid effects in the hippocampus, and showed that DHT and T induced rapid increase of CA1 dendritic spines via non-genomic signaling, including activation of several serine/threonine kinases (including MAPK, LIMK, PKA, PKC; Hatanaka et al., 2015). Also in CA3 region, T, and DHT rapidly increased thorns in stratum lucidum via MAPK and PKC, but not via PKA (Hatanaka et al., 2009).

Rapid synaptic action may require new arrangement of sex steroid receptor systems. In the hippocampus, androgen receptor (AR), and estrogen receptor (ER), classic nuclear steroid receptors, appear to be primarily located in the glutamatergic neurons (Simerly et al., 1990; Clancy et al., 1992; Brown et al., 1995; Kerr et al., 1995; Mukai et al., 2007). AR and ER are located not only in the cytoplasm and the nuclei but also within dendritic spines (Tabori et al., 2005; Mukai et al., 2007; Hatanaka et al., 2015). Therefore, classic receptors AR and ER, localized in the synaptic membrane, could act as membrane receptors, triggering rapid effects of sex steroids as indicated from many recent investigations (Milner et al., 2005; Mukai et al., 2007, 2010; Pedram et al., 2007; Hojo et al., 2008; Hasegawa et al., 2015; Levin and Hammes, 2016). Synaptic membrane localization of these receptors might be accomplished via palmitoylation of receptors (Pedram et al., 2007; Levin and Hammes, 2016)

Since adult hippocampus locally synthesizes androgen and estrogen (Hojo et al., 2004), their levels in the hippocampus are key factors for action through synaptic AR and ER. Mass-spectrometric analysis revealed that the levels of androgen and estrogen in freshly isolated male hippocampus are higher than those in plasma (Hojo et al., 2009). The levels of male hippocampal sex steroids were ~17 nM for T, ~7 nM for DHT, and ~8 nM for E2 (Hojo et al., 2009), which levels are much higher than that of circulating T (~15 nM), DHT (~0.6 nM), and E2 (~0.01 nM). Importantly, after preparation of “acute” hippocampal slices, sex steroid levels were considerably decreased to below 0.5 nM due to recovery incubation in artificial cerebrospinal fluid (ACSF) (Hojo et al., 2009, 2011; Ooishi et al., 2012a,b; Hatanaka et al., 2015). Therefore, exogenous application of sex steroids may help to elevate steroid levels back to the *in vivo* situation.

We here investigated the possible involvement of Src tyrosine kinase in rapid spine modulation of DHT, T, and E2, with considering similarity and difference in signal pathways between DHT, T, and E2 in male rats. Although Src kinase was known to be activated by androgen and E2 in prostate and breast cancer cells (Migliaccio et al., 1996, 2000), its role in hippocampal synaptic plasticity has not been well-documented.

## Materials and methods

### Animals

Young adult male Wistar rats (12 week old, 320–360 g) were purchased from Tokyo Experimental Animals Supply (Japan). All animals were maintained under a 12 h light/12 h dark cycle and free access to food and water. The experimental procedure of this research was approved by the Committee for Animal Research of Teikyo University.

### Chemicals

DHT, T and PP2 were purchased from Sigma-Aldrich (USA). Estradiol was from Wako Pure Chemicals (Japan). Lucifer Yellow was obtained from Molecular Probes (USA).

### Slice preparation

Adult male rats were deeply anesthetized by isoflurane and decapitated. Immediately after decapitation, the brain was removed from the skull and placed in ice-cold oxygenated (95% O_2_, 5% CO_2_) artificial cerebrospinal fluid (ACSF) containing (in mM): 124 NaCl, 5 KCl, 1.25 NaH_2_PO_4_, 2 MgSO_4_, 2 CaCl_2_, 22 NaHCO_3_, and 10 D-glucose (all from Wako); pH was set at 7.4. The hippocampus was then dissected and 400 μm thick transverse slices to the long axis, from the middle third of the hippocampus, were prepared with a vibratome (Dosaka, Japan). These slices were “fresh” slices without ACSF incubation. Slices were then incubated in oxygenated ACSF for 2 h (slice recovery processes) in order to obtain widely used “acute slices.”

### Imaging and analysis of dendritic spine density and morphology

#### Drug treatments and current injection of lucifer yellow

The “acute” slices (used worldwide) were incubated for 2 h with 10 nM DHT, 10 nM T, or 1 nM E2, together with 4-amino-5-(4-chlorophenyl)-7-(dimethylethyl)pyrazolo[3,4-d]pyrimidine (PP2), a Src kinase inhibitor. Slices were then fixed with 4% paraformaldehyde in PBS at 4°C overnight. Neurons within slices were visualized by an injection of Lucifer Yellow (Molecular Probes, USA) under Nikon E600FN microscope (Japan) equipped with a C2400-79H infrared camera (Hamamatsu Photonics, Japan) and with a 40× water immersion lens (Nikon, Japan).

Current injection was performed with glass electrode filled with 4% Lucifer Yellow for 2 min, using Axopatch 200B (Axon Instruments, USA). Approximately two neurons within a depth of 100–200 μm from the surface of a slice were injected with Lucifer Yellow (Duan et al., 2002).

#### Confocal laser microscopic imaging and analysis

The imaging was performed from sequential z-series scans with super-resolution confocal microscope (Zeiss LSM880; Carl Zeiss, Germany) using Airy Scan Mode, at high zoom (× 3.0) with a 63 × oil immersion lens, NA 1.4. For Lucifer Yellow, the excitation and emission wavelengths were 458 and 515 nm, respectively. For analysis of spines, three-dimensional image was reconstructed from ~30 sequential z-series sections of every 0.45 μm. The applied zoom factor (×3.0) yielded 23 pixels per 1 μm. The confocal lateral resolution was ~0.14 μm. The z-axis resolution was ~0.40 μm. Our resolution limits were regarded to be sufficient to allow the determination of the head diameter of spines in addition to the density of spines. Confocal images were deconvoluted with the measured point spread function using Processing Mode of LSM880.

The density of spines as well as the head diameter were analyzed with Spiso-3D (automated software calculating mathematically geometrical parameters of spines) developed by Bioinformatics Project of Kawato's group (Mukai et al., 2011). Spiso-3D has an equivalent capacity with Neurolucida (MicroBrightField, USA), furthermore, Spiso-3D considerably reduces human errors and experimenter labor. The single apical dendrite was analyzed separately. The spine density was calculated from the number of spines along secondary dendrites having a total length of 40–60 μm. These dendrites were present within the stratum radiatum, between 100 and 200 μm from the soma. Spine shapes were classified into three categories as follows. (1) A small-head spine, whose head diameter is smaller than 0.4 μm. (2) A middle-head spine, which has 0.4–0.5 μm spine head. (3) A large-head spine, whose head diameter is larger than 0.5 μm. These three categories were useful to distinguish different responses upon kinase inhibitor application. Small-, middle-, and large-head spines probably have different number of α-amino-3-hydroxy-5-methyl-4-isoxazolepropionic acid (AMPA) receptors, and therefore these three types of spines might have different efficiency in memory storage. The number of AMPA receptors (including GluR1 subunits) in the spine increases as the size of postsynapse increases, whereas the number of N-methyl-D-aspartate (NMDA) receptors (including NR2B subunits) might be relatively constant (Shinohara et al., 2008). Because the majority of spines (>93–95%) had a distinct head, and stubby spines and filopodia did not contribute much to overall changes, we analyzed spines having a distinct head.

### Statistical analysis

**Figure 1:**
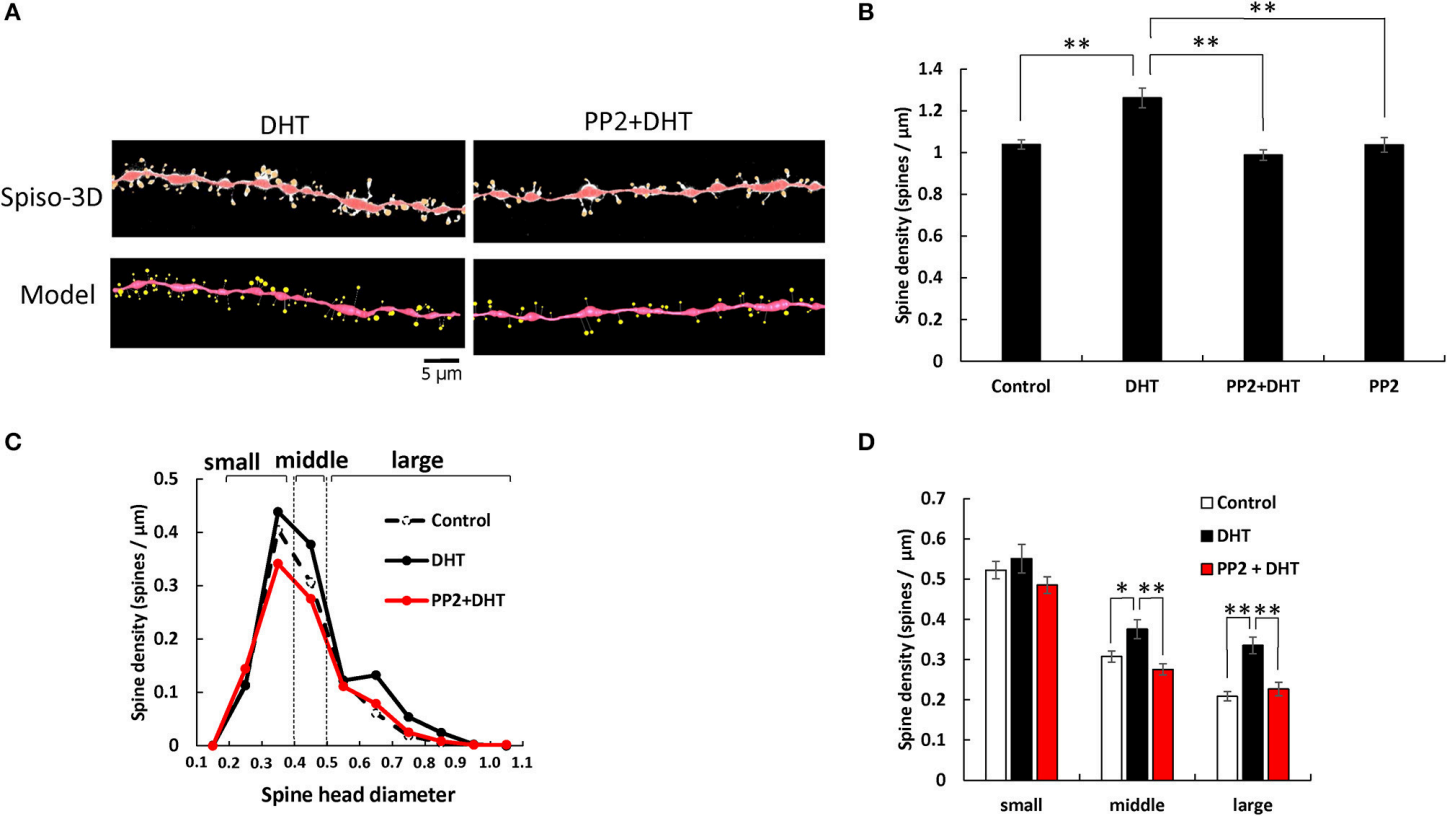
Effects of Src kinase blocker (PP2) on DHT-induced spine increase and change in morphology in hippocampal slices. **(A)** Spines were analyzed along the secondary dendrites of pyramidal neurons in the stratum radiatum of CA1 neurons. Dendrite after DHT-treatment for 2 h (DHT) and dendrite after DHT plus PP2 treatment for 2 h (PP2+DHT). (Spiso) shows the image of dendrite and spines analyzed with Spiso-3D software. Maximal intensity projections onto XY plane is shown. Traced dendrite is shown in red color and spines are indicated in yellow color. (Model) shows 3 dimensional model illustration of (Spiso) image. Bar, 5 μm. **(B)** Effect of treatments by DHT or PP2 on the total spine density in CA1 neurons. Vertical axis is the average number of spines per 1 μm of dendrite. A 2 h treatment in ACSF without drugs (Control), with 10 nM DHT (DHT), with 10 nM DHT and 10 μM PP2 (PP2 + DHT), and with PP2 only (PP2). **(C)** Histogram of spine head diameters after a 2 h treatment in ACSF without drugs (Control, black dashed line), with 10 nM DHT (black line), with 10 nM DHT and 10 μM PP2 (red line). Spines were classified into three categories depending on their head diameter, e.g., 0.2–0.4 μm as small-head spines, 0.4–0.5 μm as middle-head spines, and larger than 0.5 μm as large-head spines. Vertical axis is the number of spines per 1 μm of dendrite. **(D)** Density of three subtypes of spines. Abbreviations are same as in **(B)**. Vertical axis is the number of spines per 1 μm of dendrite. From left to right, small-head spines (small), middle-head spines (middle), and large-head spines (large) type. ACSF without drugs (Control, open column), DHT (black column), PP2 + DHT (red column). Vertical axis is the number of spines per 1 μm of dendrite. Results are represented as mean ± SEM. Statistical significance yielded *P < 0.05, **P < 0.01 vs. DHT sample. For DHT, PP2+DHT, and PP2 only treatments, we investigated, 50 dendrites with 2,300–2,700 spines from 3 rats, 12 slices, and 30 neurons. For control, we used 80 dendrites with ~4,000 spines from 6 rats, 24 slices and 50 neurons.

**Figure 2:**
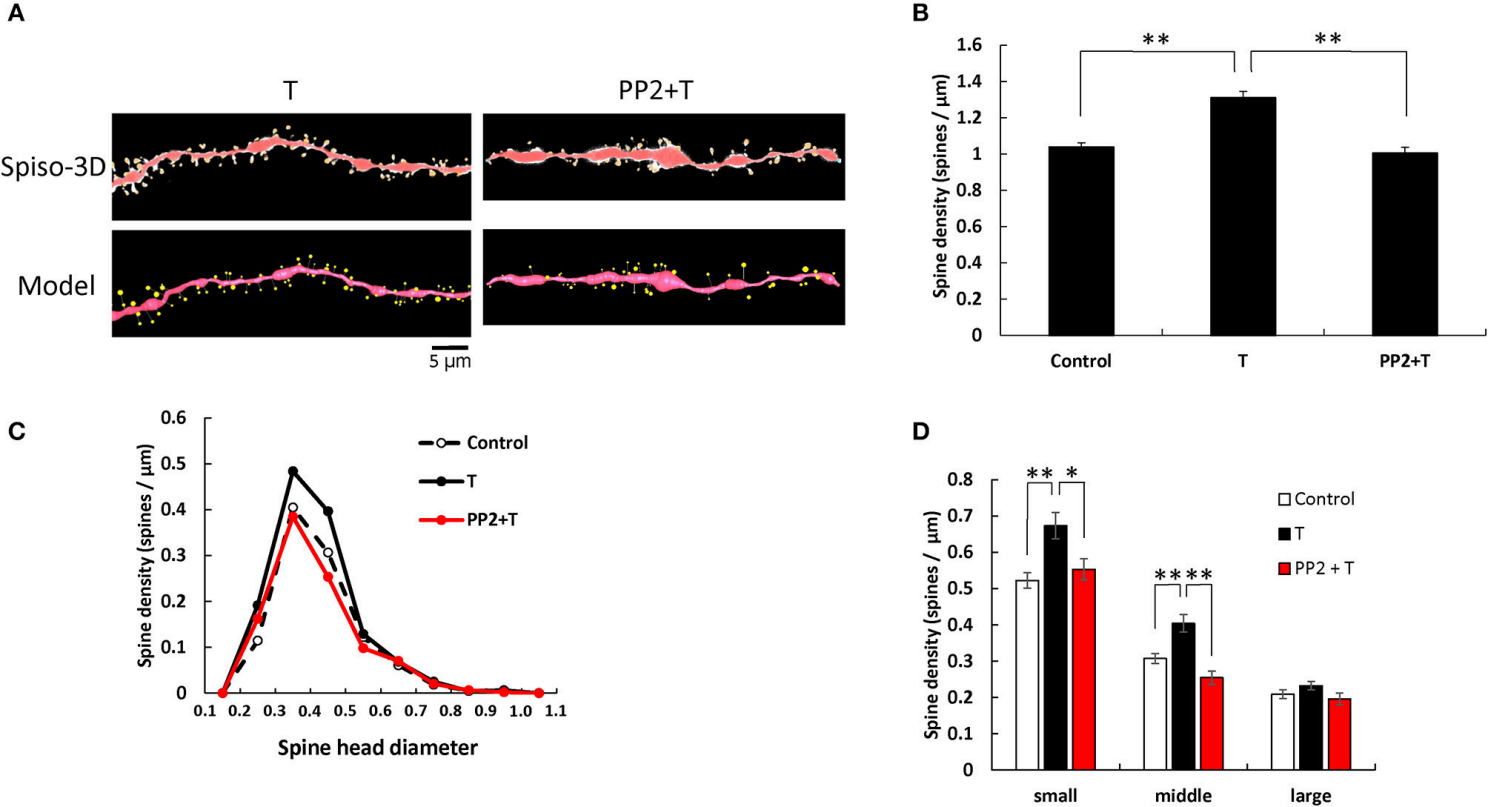
Effects of Src kinase blocker on T-induced spine increase and change in morphology in hippocampal slices. **(A)** Spines were analyzed along the secondary dendrites of pyramidal neurons in the stratum radiatum of CA1 neurons as in Figure 1. Dendrite after T-treatment for 2 h (T) and dendrite after T plus PP2 treatment for 2 h (PP2+T). (Spiso) shows the image of dendrite and spines analyzed with Spiso-3D software. Maximal intensity projections onto XY plane is shown. Traced dendrite is shown in red color and spines are indicated in yellow color. (Model) shows 3 dimensional model illustration of (Spiso) image. Bar, 5 μm. **(B)** Effect of treatments by T or PP2 on the total spine density in CA1 neurons. Vertical axis is the average number of spines per 1 μm of dendrite. A 2 h treatment in ACSF without drugs (Control), with 10 nM T (T), with 10 nM T and 10 μM PP2 (PP2 + T). **(C)** Histogram of spine head diameters after a 2 h treatment in ACSF without drugs (Control, black dashed line), with 10 nM T (black line), with 10 nM T and 10 μM PP2 (PP2 + T, red line). **(D)** Density of three subtypes of spines. Abbreviations are same as in **(B)**. From left to right, small-head spines (small), middle-head spines (middle), and large-head spines (large) type. ACSF without drugs (Control, open column), T (black column), PP2 + T (red column). Results are represented as mean ± SEM. Statistical significance yielded *P < 0.05, **P < 0.01 vs. T sample. For T and PP2 + T treatments, we investigated 50 dendrites with 2300–2700 spines from 3 rats, 12 slices, 30 neurons. For control, we used 80 dendrites with ~4,000 spines from 6 rats, 24 slices, and 50 neurons.

**Figure 3:**
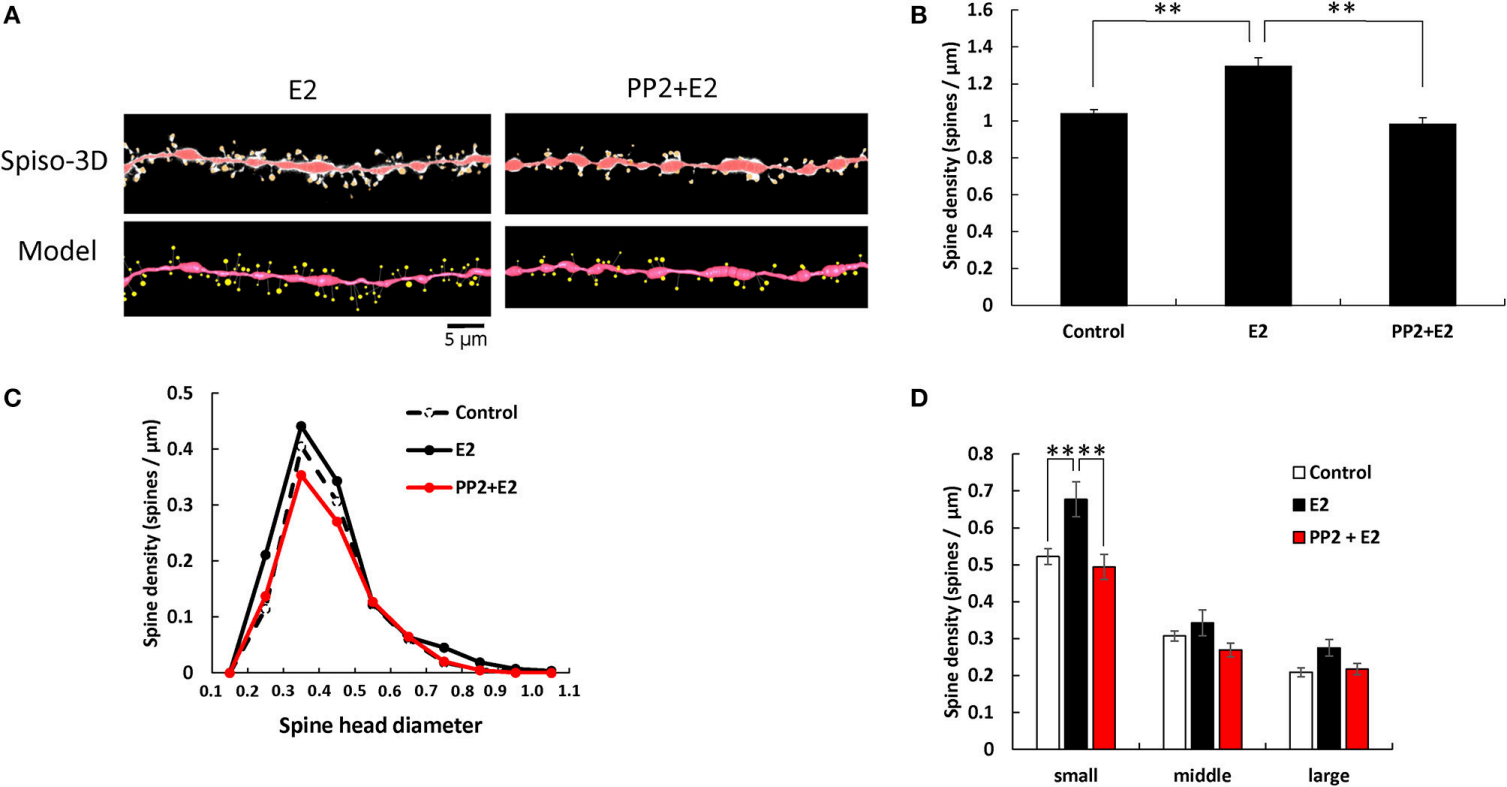
Effects of Src kinase blocker on E2-induced spine increase and change in morphology. **(A)** Spines were analyzed along the secondary dendrites of pyramidal neurons in the stratum radiatum of CA1 neurons as in Figure 1. Dendrite after E2-treatment for 2 h (E2) and dendrite after E2 plus PP2 treatment for 2 h (PP2+E2). (Spiso) shows the image of dendrite and spines analyzed with Spiso-3D software. Maximal intensity projections onto XY plane is shown. Traced dendrite is shown in red color and spines are indicated in yellow color. (Model) shows 3 dimensional model illustration of (Spiso) image. Bar, 5 μm. **(B)** Effect of treatments by E2 and PP2 on the total spine density. Vertical axis is the average number of spines per 1 μm of dendrite. A 2 h treatment in ACSF without drugs (Control), with 1 nM E2 (E2), and with 1 nM E2 and 10 μM PP2 (PP2 + E2). **(C)** Histogram of spine head diameters. A 2 h treatment in ACSF without drugs (Control, dashed line), with E2 (black line), with E2 + PP2 (red line). **(D)** Density of three subtypes of spines. From left to right, small-head spines (small), middle-head spines (middle), and large-head spines (large) type. In each group, control (open column), E2 (black column), and PP2+E2 (red column). Results are represented as mean ± SEM. Statistical significance was defined as *p < 0.05, **p < 0.01 vs. E2 sample. For E2 and PP2+E2 treatments, we investigated 50 dendrites with 2300–2700 spines from 3 rats, 12 slices, and 30 neurons. For control, we used 80 dendrites with ~4,000 spines from 6 rats, 24 slices, and 50 neurons.

**Figure 4:**
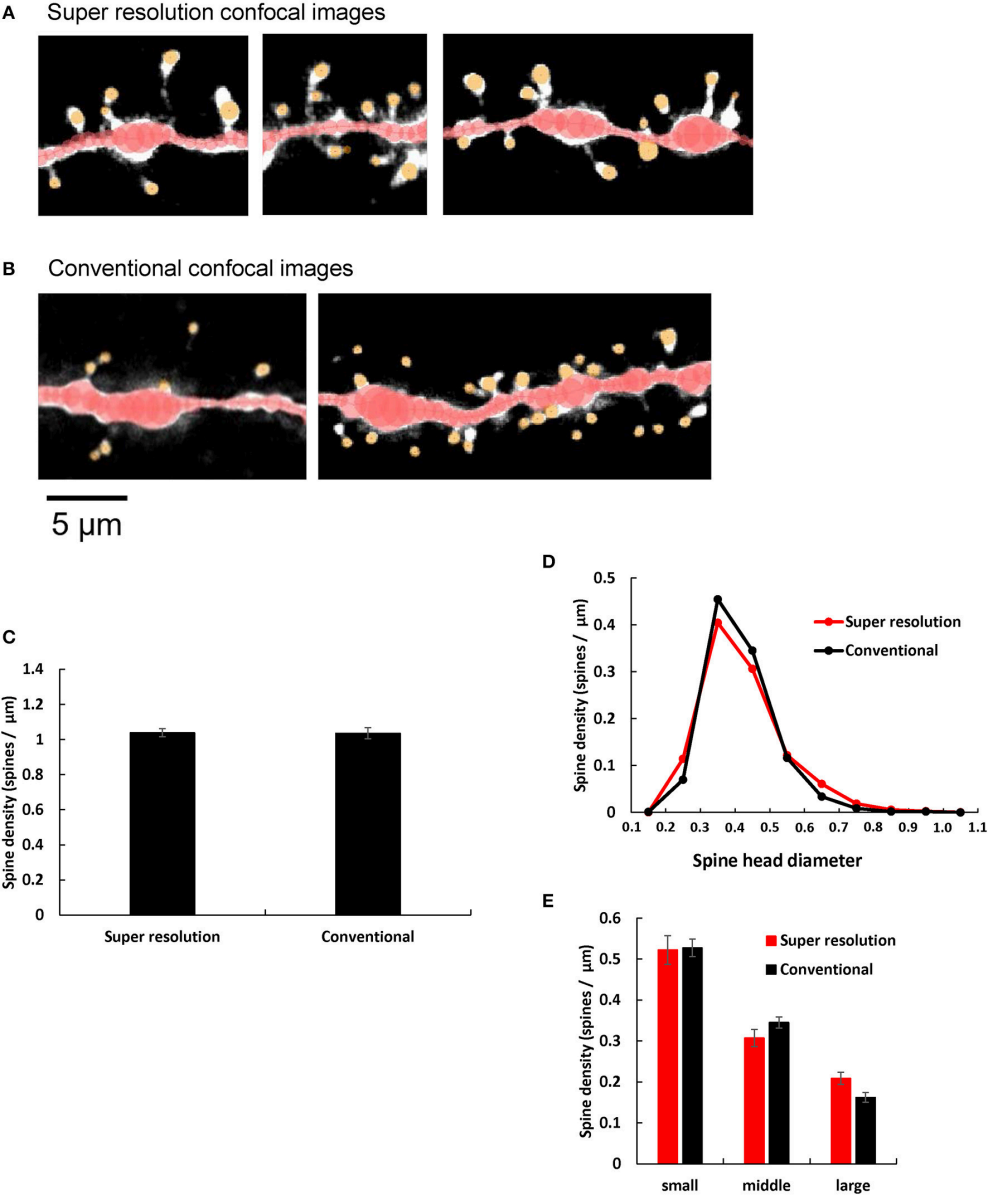
Comparison of spine images obtained by super resolution confocal microscopy and conventional confocal microscopy. **(A,B)** Typical dendrites with spines of CA1 neurons are shown after analysis with Spiso-3D software. Maximal intensity projections onto XY plane are shown. Traced dendrites are shown in red color and spines are indicated in yellow color. Images are obtained with **(A)** Super resolution confocal microscopy and **(B)** Conventional confocal microscopy. Bar, 5 μm. **(C)** Comparison of the total spine density of control dendrites (without steroid supplementation) obtained with super resolution confocal microscopy (Super resolution) and Conventional confocal microscopy (Conventional). Vertical axis is the average number of spines per 1 μm of dendrite. **(D)** Histogram of spine head diameters of control. Super resolution (red line), and Conventional (black line). **(E)** Density of three subtypes of spines of control dendrites. From left to right, small-head spines (small), middle-head spines (middle), and large-head spines (large). In each group, Super resolution (red column), and Conventional (black column). Abbreviations are same as in **(C)**. Results are represented as mean ± SEM. No statistical significance was observed. For Super resolution, we investigated 80 dendrites with ~4,000 spines, 6 rats, 24 slices, and 50 neurons. For Conventional, we used 40 dendrites with ~2,000 spines from 4 rats, 10 slices, and 21 neurons.

Drug-treated dendrite images were used for spine analysis, and typical images were shown in Figures 1–**4**. Each dendrite has ~50 μm in length including ~50 spines. For statistical analysis, we employed two-way ANOVA, followed by Tukey-Kramer multiple comparison's test. For each steroid application analysis, we used ~50 dendrites with 2,300–2,700 spines obtained from 3 rats, 12 slices, 30 neurons. For control dendrite analysis without steroid application, we used 80 dendrites with ~4,000 spines from 6 rats, 24 slices, 50 neurons.

## Results

We investigated the involvement of Src protein kinase in the modulation effects of DHT, T, and E2 on spinogenesis. Dendritic spine imaging was performed for Lucifer Yellow-injected glutamatergic neurons in acute hippocampal slices of male rats. We analyzed secondary branches of the apical dendrites located 100–200 μm distant from the pyramidal cell body around the middle of the stratum radiatum of CA1 region.

### Analysis of spine head diameter as well as the total spine density

The morphological changes in spine head diameter induced after 2 h treatments of drugs were analyzed. Since observing the total spine density cannot describe well the complicated different kinase effects, the changes in spine head diameter distribution were also analyzed. Because the majority of spines (>93–95%) had distinct heads and necks, and stubby spines and filopodia did not contribute much to overall changes (<5–7%), we analyzed spines having distinct heads. We classified these spines with clear heads into three categories based on their head diameter, e.g., 0.2–0.4 μm as small-head spines, 0.4–0.5 μm as middle-head spines, and larger than 0.5 μm as large-head spines.

Statistical analyses based on classification of the spines into three categories were performed. In control slices (without sex steroids supplementation), the spine density was 0.52 spines/μm for small-head spines, 0.30 spines/μm for middle-head spines, and 0.20 spines/μm for large-head spines (Figure 1). In order to investigate intracellular signaling pathways of kinases involved in the sex steroid-induced spinogenesis, here, we analyzed the contribution of Src protein kinase by blocking Src kinase with a selective inhibitor, PP2.

### DHT effects and src kinase blocking

The treatments with DHT and PP2 induced significant changes in the total spine density (*F* = 20.46, *p* < 0.0001, two-way ANOVA). After 2 h treatment with 10 nM DHT, the total spine density was significantly increased to 1.26 spines/μm from the control density of 1.04 spines/μm (*p* < 0.0001 for control vs. DHT, Tukey–Kramer multiple comparison's test) (see Figure 1). This increase in the total spine density was suppressed by blocking of Src kinase through co-incubation of 10 nM DHT and 10 μM PP2 (*p* < 0.0001 for DHT vs. PP2+ DHT, Tukey–Kramer multiple comparison's test).

From spine head diameter analysis, after 2 h treatments with DHT, the density of middle- and large-head spines significantly increased (*p* = 0.0304 for middle-head and *p* < 0.0001 for large-head, for control vs. DHT, Tukey–Kramer), while the density of small-head spines was not significantly altered. Blocking Src kinase by PP2 abolished the DHT effects on the dendritic spine densities, by decreasing the density of the middle-head spines and large-head spines (*p* = 0.0016 for middle and *p* = 0.0004 for large-head, for DHT vs. PP2+ DHT, Tukey–Kramer), while significant changes in the small-head spines did not occur (Figures 1B–D).

Note that, only PP2 did not significantly affect the total spine density, implying that the observed inhibitory effects are not due to simple non-specific effects by blockers (Figure 1B).

### T effects and Src kinase blocking

T and PP2 treatments induced significant changes in the total spine density (*F* = 25.68, *p* < 0.0001, two-way ANOVA). The total spine density was significantly increased to 1.31 spines/μm through 2 h incubation with 10 nM T (*p* < 0.0001 for control vs. T, Tukey–Kramer) (see Figure 2). This increase in the total spine density was suppressed by blocking Src kinase through co-incubation of 10 nM T and 10 μM PP2 (*p* < 0.0001 for T vs. PP2+T, Tukey–Kramer) (Figure 2B).

From spine head diameter analysis, upon treatments with T, the density of small- and middle-head spines significantly increased (*p* = 0.0004 for small-head and *p* = 0.0005 for middle-head, Tukey–Kramer), while the density of large-head spines was not significantly altered. Inhibition of Src kinase abolished the effect of T on the spine density, by decreasing the density of small-and middle-head spines (*p* = 0.0248 for small-head and *p* < 0.0001 for middle-head, Tukey–Kramer), while there were no significant changes in large-head spines (Figures 2B–D).

### E2 effects and Src kinase blocking

E2 and PP2 treatments induced significant changes in the total spine density (*F* = 19.71, *p* < 0.0001, two-way ANOVA). After 2 h treatments with 1 nM E2, the total spine density was significantly increased to 1.29 spines/μm (*p* < 0.0001 for control vs. E2, Tukey–Kramer) (Figure 3). This increase in the total spine density was suppressed by blocking of Src kinase through co-incubation of 1 nM E2 and 10 μM PP2 (*p* < 0.0001 for E2 vs. PP2+ E2, Tukey–Kramer) (Figure 3A).

From spine head diameter analysis, treatments with E2 significantly increased the density of small-head spines (*p* = 0.0079, Tukey–Kramer), while the density of middle- and large-head spines was not significantly altered. Blocking Src kinase by PP2 suppressed the effects of E2, by decreasing the density of small-head spines (*p* = 0.0075, Tukey–Kramer), while significant change was not observed in middle- and large-head spines (Figures 3B–D).

### Improvement of image analysis with super resolution confocal microscopy

Airy scan mode of LSM880 super resolution confocal microscopy is equipped with 32 channel area detectors with honeycomb arrangement in the back of confocal pinhole. With these detectors we can directly measure the main part of the point spread function with which we perform deconvolution of confocal images. Therefore, the resultant final spine images were much clearer particularly for neck images with super resolution confocal microscopic imaging (Figure 4A) than those with conventional confocal microscopic imaging (Figure 4B) which uses blind deconvolution method with AutoDeblur software (AutoQuant, USA). Particularly necks of spines were clearly observed in Airy Scan mode, while sometimes spine heads spatially isolated from dendrites, without visible necks which connect spine heads to the dendritic shafts, were observed with conventional confocal microscopic imaging (Figure 4B). Identification of necks is critical in classification of spine types between stubby type (without neck) and spines with necks (thin and mushroom types).

We quantitatively compared control dendritic spines (without steroid supplementation) obtained from super resolution confocal microscopic analysis (LSM880) with those obtained from conventional confocal microscopic analysis (with Zeiss PASCAL confocal microscopy) (Hasegawa et al., 2015). We observed no significant difference in spine densities within experimental error concerning not only the total spine density but also the head diameter distribution, between these two confocal microscopic analyses. (Figures 4C–E). However, the ratio of large-head spine population and middle-head spine population was little bit higher in super resolution than conventional confocal analysis.

## Discussion

We consider and discuss about Src kinase dependent signaling mechanisms in non-genomic modulation of sex steroid-induced dendritic spinogenesis.

### Src kinase-mediated signaling with sex steroids treatments in peripheral tissues

Involvement of Src kinase (nonreceptor tyrosine kinase) in non-genomic rapid signaling upon androgen and estrogen stimulation has been extensively investigated in non-neuronal cells, including prostate cancer cells (e.g., LNCaP cells) (Migliaccio et al., 2000), breast cancer cells (e.g., MCF-7 cells) (Migliaccio et al., 1996), epithelial cells (Castoria et al., 2004), and Sertoli cells (Cheng et al., 2007).

Both T and E2 induced complex formation of AR, ERβ and Src kinase in LNCaP (Migliaccio et al., 2000). E2-induced complex formation of ERα with Src kinase was observed in MCF-7 cells (Migliaccio et al., 2000). Src kinase phosphorylated Erk MAPK in MCF-7 cells (Migliaccio et al., 1996). Upon T stimulation in Sertoli cells, association of AR with Src kinase occurred, leading to activation of Erk MAPK (Cheng et al., 2007).

**Figure 5:**
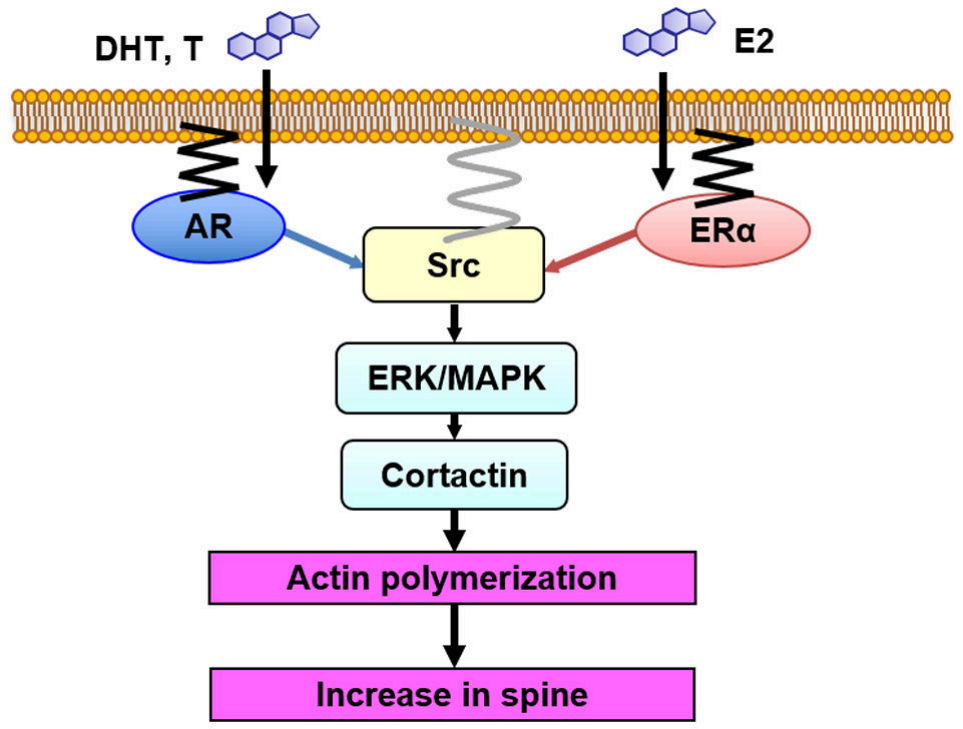
Model illustration of Src kinase-mediated signaling in non-genomic effects of sex steroids on spinogenesis. DHT, T and E2 bind to synaptic receptors (AR and ER) within spines. Then, Src kinase may form complex with AR and ER, resulting in activation of Src kinase. Erk MAPK is then activated, leading to phosphorylation of cortactin, resulting in actin polymerization and new spine formation. AR and ER are anchored to the membrane via palmitoylation. Src kinase is localized to the membrane via myristoylation.

Taken together, upon stimulation of T or E2, AR or ER may form complex with Src kinase, leading to activation of MAPK in prostate cancer cells, breast cancer cells, or other gonadal tissues (Foradori et al., 2008). Androgen-induced Ca influx may be a trigger of these events in these cells (Rusanescu et al., 1995; Foradori et al., 2008). Src kinase activation is induced by dephosphorylation of tyrosine residue, and this might occur within complexes of AR, ER, and Src kinase, via AR binding to SH3 domain or via ER binding to SH2 domain (Migliaccio et al., 2000). Note that Src kinase is anchored to the membrane via myristoylation (Figure 5; Kim et al., 2017).

### Src kinase-mediated signaling with sex steroid treatments in neurons

We observed association of Src kinase, Erk MAPK, and ERα with post synaptic density (PSD), using Western blotting of PSD fractions prepared from male rat hippocampi, implying the spine membrane binding of Src kinase, MAPK, and ER (Mukai et al., 2007). Therefore, from the current results in combination with the results from endocrine tissues (see section Src Kinase-Mediated Signaling With Sex Steroids Treatments in Peripheral Tissues), we can speculate that Src kinase-mediated signaling, triggered by sex steroids in hippocampal spines, may occur as follows: upon T, DHT, and E2 binding to their synaptic receptors (AR and ERα), these receptors activate Src kinase via conformational change of Src, leading to phosphorylation of Erk MAPK (Figure 5).

Until recently, however, inadequate amounts of investigations had been accumulated for Src kinase signaling upon androgen and estrogen stimulation in central nervous systems. In one of a few reports, Src tyrosine kinase involvement was suggested from PP2-induced suppression of E2-enhanced LTP in acute hippocampal slices (Bi et al., 2000). Chronic DHT-treatment for 24 h elevated Ca concentrations in the endoplasmic reticulum of primary hippocampal neurons, but this might not directly contribute to non-genomic actions (Foradori et al., 2007).

On the other hand, downstream signaling of Src kinase, from MAPK to spine increase, has been extensively studied in neurons (see section MAPK-Mediated Signaling, Downstream of Src Kinase in Spinogenesis).

### MAPK-mediated signaling, downstream of Src kinase in spinogenesis

Erk MAPK (serine/threonine kinase) was involved in the rapid non-genomic effects on CA1 hippocampal spinogenesis through DHT, T, and E2 treatments (Hasegawa et al., 2015; Hatanaka et al., 2015). These results were obtained by using MAPK inhibitors, U0126 and PD98059. E2-induced rapid CA1 spinogenesis via Erk MAPK was found in the male and female hippocampus (Mukai et al., 2007; Phan et al., 2011; Luine and Frankfurt, 2012). *In vivo* E2 infusion induced CA1 spinogenesis as well as phosphorylation of Erk MAPK in female ovariectomized (OVX) mouse hippocampus (Frick et al., 2015; Tuscher et al., 2016). T and DHT individually phosphorylated Erk MAPK within 1 h, leading to neuroprotection in primary cultured hippocampal neurons (Nguyen et al., 2005).

Since both steroid receptors (AR and ER), Src kinase and MAPK are present in dendritic spines, an efficient coupling between these proteins could occur in spines, resulting in activation of Src kinase, followed by activation of Erk MAPK (Figure 5) (Milner et al., 2005; Tabori et al., 2005; Mukai et al., 2007; Hojo et al., 2008; Hatanaka et al., 2015).

In spinogenesis, the target of Erk MAPK may be cortactin. Erk MAPK phosphorylates cortactin which is associated with actin (MacQueen et al., 2003). Cortactin interacts with both F-actin and actin-related protein (Arp) 2/3 complex as well as scaffold protein Shank (Weed et al., 1998; Campbell et al., 1999), leading to promotion of actin fiber remodeling within spines. Therefore, it is possible that DHT, T, and E2 might modulate spines via cortactin-actin pathway. Cortactin has multiple phosphorylation sites, such as Ser^113^, Ser^405^, and Ser^418^, which are putative phosphorylation sites of MAPK (Campbell et al., 1999). Phosphorylation of cortactin triggered by DHT, T, and E2 may promote assembly of actin cytoskeleton, which either leads to increasing spines or modulating the spine morphology (Hering and Sheng, 2003). The involvement of cortactin in androgen-induced modulation of spines is suggested from the results that AR inhibitor induced actin depolymerization via tyrosine phosphorylation of cortactin (Anahara et al., 2006).

### Localization of AR and ER in spines

The involvement of classic AR in androgen-induced rapid action was indicated from suppressing effects of hydroxyflutamide, a specific inhibitor of AR, on androgen-induced spinogenesis in acute slices of male rat hippocampus (Hatanaka et al., 2015). The AR immunostaining was localized in CA1 neurons with optical microscopic analysis, and synaptic localization of AR was indicated with immuno-electron microscopic analysis (Tabori et al., 2005). Western blot analysis showed the AR expression in PSD fractions as well as in nuclear and cytoplasmic fractions, which implies that AR localized in the PSD can participate in the T- and DHT-induced spine increase (Hatanaka et al., 2015). These results suggest that AR localized in spines could mediate androgen-induced rapid spine increase through activation of Src kinase and MAPK.

The involvement of classic ER in rapid E2 action was indicated from suppressing effects of ICI182,780 (ICI), a specific antagonist of both ER, on E2-induced spinogenesis in male hippocampal slices (Mukai et al., 2007; Hasegawa et al., 2015). Involvement of ERα or ERβ in rapid spinogenesis was also examined by using estrogen receptor agonists. ERα agonist, (propyl-pyrazole-trinyl) tris-phenol (PPT), unlike ERβ agonist such as (4-hydroxyphenyl)-propionitrile (DPN), acutely increased the density of spines in hippocampal CA1 neurons in male rat slices (Hasegawa et al., 2015) and OVX female mice (Phan et al., 2011). These results support the exclusive involvement of ERα in rapid signaling. ERαKO mice and ERβKO mice studies further confirmed the involvement of ERα, but not ERβ, in the rapid E2 signaling (Murakami et al., 2015).

Expression of ERα in neurons of CA1 in rat and mouse hippocampus was demonstrated by immunostaining with purified antibody RC-19 (Mukai et al., 2007). ERα was located in spines in addition to nuclei/cytoplasm, as revealed by the immunogold electron-microscopic analyses (Mukai et al., 2007). Spine membrane localization of AR and ER may be accomplished via palmitoylation of receptors (Figure 5), as judged from recent studies, including the finding that some populations of ERα and ERβ were plasma membrane-bound in cultured breast cancer cells MCF-7 (Pedram et al., 2006), and they were anchored via palmitoylation (Pedram et al., 2007).

### Difference between DHT, T, and E2 in rapid effects on spine density and morphology in CA1

Although treatments with DHT, T, and E2 increased the total spine density to almost the same level, three subclass analysis showed clear differences between DHT, T, and E2 in modulation of the spine morphology of hippocampal CA1 neurons (Figures 1–3). DHT treatments considerably increased the density of large-head spines. On the other hand, all the small- and middle-head spines were increased with T treatments, whereas E2 increased only small-head spines. Since large-head spines have significantly more AMPA receptors than small-head spines (Shinohara et al., 2008), DHT might increase synapses with higher memory storage capacity than T and E2.

The T-effect is not dependent on the conversion from T to E2, and neither from T to DHT. This is concluded from the fact that T-effect was not blocked by inhibition of P450arom (E2 synthetase) and 5α-reductase (DHT synthetase) (Hatanaka et al., 2015). Moreover, the effect of T was blocked by AR antagonist. T-effect must be therefore directly mediated by AR, and not mediated by E2.

### Difference in sex steroid levels between acute hippocampal slices and *in vivo* hippocampus

Sex steroid levels in the hippocampus play a key role for rapid action through synaptic AR and ER as modulators of synaptic plasticity. The average concentrations of male rat hippocampal DHT, T, and E2 *in vivo* were determined to be ~7, 17, and 8 nM, respectively, in freshly isolated hippocampi, with mass-spectrometric analysis (Hojo et al., 2008, 2009) (Okamoto et al., 2012). These hippocampal sex steroid levels are significantly higher than plasma sex steroid levels, due to hippocampal synthesis of sex steroids, in addition to penetration of T (~15 nM in plasma) to the hippocampus via blood circulation (Hojo et al., 2009). Interestingly, E2 level in the male hippocampus (~8 nM) is higher than that in female (0.6–4.3 nM), due to conversion of high level T into E2 within the hippocampus by P450arom (Kato et al., 2013). Importantly in “acute” slices (used for current analysis of synaptic plasticity), the levels of DHT, T, and E2 decreased to below 0.5 nM due to the slice recovery incubation for 2 h with ACSF after fresh slice preparations (Hojo et al., 2009, 2011; Ooishi et al., 2012a,b). In the current study, the exogenous application of 10 nM DHT, 10 nM T, and 1 nM E2 individually was used to rapidly elevate the hippocampal sex steroid levels from the steroid-depleted levels in “acute” slice (<0.5 nM), to nearly the endogenous levels of male rat (Ooishi et al., 2012a), resulting in rapid action of sex steroids.

### Toward *in vivo* analysis of sex steroid effects on spine modulation

The currently obtained knowledge encourages analysis of *in vivo* mechanisms of rapid sex steroid actions on spines. Recent *in vivo* investigations implied that E2 treatments with s.c. injection rapidly (within 30–40 min) increased the spine density in CA1 hippocampal neurons of OVX female mice (Phan et al., 2011, 2012; Jacome et al., 2016; Tuscher et al., 2016). These *in vivo* investigations can be interpreted that E2 supplementation recovered the spine density, because OVX surgery once decreased spine density as well as declined hippocampal E2 level due to depletion of circulating E2 (Kato et al., 2013). Such approaches in combination with kinase inhibitors may be useful to analyze *in vivo* molecular mechanisms of rapid synaptic modulation by E2 in female rodents. Concerning male rodents, approaches with castration followed by rapid androgen supplementation could be promising to analyze mechanisms of rapid modulation effects of androgen on spines, because castration decreases hippocampal T and DHT as well as the spine density.

## Author contributions

SK: conceived and designed the study; MS, JK, and AK: conducted the experiments and analysis of the data; SK: wrote the manuscript. All authors provided feedback on the manuscript.

### Conflict of interest statement

The authors declare that the research was conducted in the absence of any commercial or financial relationships that could be construed as a potential conflict of interest.

## References

[B1] Anahara R. Toyama Y. Maekawa M. Yoshida M. Kai M.. (2006). Anti-estrogen ICI 182.780 and anti-androgen flutamide induce tyrosine phosphorylation of cortactin in the ectoplasmic specialization between the Sertoli cell and spermatids in the mouse testis. Biochem. Biophys. Res. Commun. 346, 276–280. 10.1016/j.bbrc.2006.05.125 16756952

[B2] Bi R. Broutman G. Foy M. R. Thompson R. F. Baudry M. (2000). The tyrosine kinase and mitogen-activated protein kinase pathways mediate multiple effects of estrogen in hippocampus. Proc. Natl. Acad. Sci. U.S.A. 97, 3602–3607. 10.1073/pnas.97.7.3602 10725383 PMC16286

[B3] Bi R. Foy M. R. Vouimba R. M. Thompson R. F. Baudry M. (2001). Cyclic changes in estradiol regulate synaptic plasticity through the MAP kinase pathway. Proc. Natl. Acad. Sci. U.S.A. 98, 13391–13395. 10.1073/pnas.241507698 11687663 PMC60881

[B4] Brown T. J. Sharma M. Heisler L. E. Karsan N. Walters M. J. MacLusky N. J. (1995). *In vitro* labeling of gonadal steroid hormone receptors in brain tissue sections. Steroids 60, 726–737. 10.1016/0039-128X(95)00107-2 8585096

[B5] Campbell D. H. Sutherland R. L. Daly R. J. (1999). Signaling pathways and structural domains required for phosphorylation of EMS1/cortactin. Cancer Res. 59, 5376–5385. 10537323

[B6] Castoria G. Lombardi M. Barone M. V. Bilancio A. Di Domenico M.. (2004). Rapid signalling pathway activation by androgens in epithelial and stromal cells. Steroids 69, 517–522. 10.1016/j.steroids.2004.05.001 15288763

[B7] Cheng J. Watkins S. C. Walker W. H. (2007). Testosterone activates mitogen-activated protein kinase via Src kinase and the epidermal growth factor receptor in sertoli cells. Endocrinology 148, 2066–2074. 10.1210/en.2006-1465 17272394

[B8] Clancy A. N. Bonsall R. W. Michael R. P. (1992). Immunohistochemical labeling of androgen receptors in the brain of rat and monkey. Life Sci. 50, 409–417. 10.1016/0024-3205(92)90375-Y 1734159

[B9] Duan H. Wearne S. L. Morrison J. H. Hof P. R. (2002). Quantitative analysis of the dendritic morphology of corticocortical projection neurons in the macaque monkey association cortex. Neuroscience 114, 349–359. 10.1016/S0306-4522(02)00305-6 12204204

[B10] Foradori C. D. Weiser M. J. Handa R. J. (2008). Non-genomic actions of androgens. Front. Neuroendocrinol. 29, 169–181. 10.1016/j.yfrne.2007.10.005 18093638 PMC2386261

[B11] Foradori C. D. Werner S. B. Sandau U. S. Clapp T. R. Handa R. J. (2007). Activation of the androgen receptor alters the intracellular calcium response to glutamate in primary hippocampal neurons and modulates sarco/endoplasmic reticulum calcium ATPase 2 transcription. Neuroscience 149, 155–164. 10.1016/j.neuroscience.2007.06.054 17870249

[B12] Foy M. R. Xu J. Xie X. Brinton R. D. Thompson R. F. Berger T. W. (1999). 17beta-estradiol enhances NMDA receptor-mediated EPSPs and long-term potentiation. J. Neurophysiol. 81, 925–929. 10.1152/jn.1999.81.2.925 10036289

[B13] Frick K. M. (2015). Molecular mechanisms underlying the memory-enhancing effects of estradiol. Horm. Behav. 74, 4–18. 10.1016/j.yhbeh.2015.05.001 25960081 PMC4573242

[B14] Frick K. M. Kim J. Tuscher J. J. Fortress A. M. (2015). Sex steroid hormones matter for learning and memory: estrogenic regulation of hippocampal function in male and female rodents. Learn. Mem. 22, 472–493. 10.1101/lm.037267.114 26286657 PMC4561402

[B15] Gould E. Woolley C. S. Frankfurt M. McEwen B. S. (1990). Gonadal steroids regulate dendritic spine density in hippocampal pyramidal cells in adulthood. J. Neurosci. 10, 1286–1291. 10.1523/JNEUROSCI.10-04-01286.1990 2329377 PMC6570209

[B16] Grassi S. Tozzi A. Costa C. Tantucci M. Colcelli E.. (2011). Neural 17beta-estradiol facilitates long-term potentiation in the hippocampal CA1 region. Neuroscience 192, 67–73. 10.1016/j.neuroscience.2011.06.078 21749911

[B17] Hajszan T. MacLusky N. J. Leranth C. (2008). Role of androgens and the androgen receptor in remodeling of spine synapses in limbic brain areas. Horm. Behav. 53, 638–646. 10.1016/j.yhbeh.2007.12.007 18262185 PMC2408746

[B18] Hasegawa Y. Hojo Y. Kojima H. Ikeda M. Hotta K.. (2015). Estradiol rapidly modulates synaptic plasticity of hippocampal neurons: involvement of kinase networks. Brain Res. 1621, 147–161. 10.1016/j.brainres.2014.12.056 25595055

[B19] Hatanaka Y. Hojo Y. Mukai H. Murakami G. Komatsuzaki Y.. (2015). Rapid increase of spines by dihydrotestosterone and testosterone in hippocampal neurons: dependence on synaptic androgen receptor and kinase networks. Brain Res. 1621, 121–132. 10.1016/j.brainres.2014.12.011 25511993

[B20] Hatanaka Y. Mukai H. Mitsuhashi K. Hojo Y. Murakami G.. (2009). Androgen rapidly increases dendritic thorns of CA3 neurons in male rat hippocampus. Biochem. Biophys. Res. Commun. 381, 728–732. 10.1016/j.bbrc.2009.02.130 19254689

[B21] Hering H. Sheng M. (2003). Activity-dependent redistribution and essential role of cortactin in dendritic spine morphogenesis. J. Neurosci. 23, 11759–11769. 10.1523/JNEUROSCI.23-37-11759.2003 14684878 PMC6740953

[B22] Hojo Y. Hattori T. A. Enami T. Furukawa A. Suzuki K.. (2004). Adult male rat hippocampus synthesizes estradiol from pregnenolone by cytochromes P45017alpha and P450 aromatase localized in neurons. Proc. Natl. Acad. Sci. U.S.A. 101, 865–870. 10.1073/pnas.2630225100 14694190 PMC321772

[B23] Hojo Y. Higo S. Ishii H. Ooishi Y. Mukai H.. (2009). Comparison between hippocampus-synthesized and circulation-derived sex steroids in the hippocampus. Endocrinology 150, 5106–5112. 10.1210/en.2009-0305 19589866

[B24] Hojo Y. Higo S. Kawato S. Hatanaka Y. Ooishi Y.. (2011). Hippocampal synthesis of sex steroids and corticosteroids: essential for modulation of synaptic plasticity. Front. Endocrinol. 2:43. 10.3389/fendo.2011.00043 22701110 PMC3356120

[B25] Hojo Y. Murakami G. Mukai H. Higo S. Hatanaka Y.. (2008). Estrogen synthesis in the brain–role in synaptic plasticity and memory. Mol. Cell Endocrinol. 290, 31–43. 10.1016/j.mce.2008.04.017 18541362

[B26] Jacome L. F. Barateli K. Buitrago D. Lema F. Frankfurt M. Luine V. N. (2016). Gonadal hormones rapidly enhance spatial memory and increase hippocampal spine density in male rats. Endocrinology 157, 1357–1362. 10.1210/en.2015-1959 26844375 PMC4816741

[B27] Kato A. Hojo Y. Higo S. Komatsuzaki Y. Murakami G.. (2013). Female hippocampal estrogens have a significant correlation with cyclic fluctuation of hippocampal spines. Front. Neural. Circuits 7:149. 10.3389/fncir.2013.00149 24151456 PMC3798982

[B28] Kerr J. E. Allore R. J. Beck S. G. Handa R. J. (1995). Distribution and hormonal regulation of androgen receptor (AR) and AR messenger ribonucleic acid in the rat hippocampus. Endocrinology 136, 3213–3221. 10.1210/endo.136.8.7628354 7628354

[B29] Kim S. Alsaidan O. A. Goodwin O. Li Q. Sulejmani E. Han Z.. (2017). Blocking myristoylation of src inhibits its kinase activity and suppresses prostate cancer progression. Cancer Res. 77, 6950–6962. 10.1158/0008-5472.CAN-17-0981 29038344 PMC5732839

[B30] Levin E. R. Hammes S. R. (2016). Nuclear receptors outside the nucleus: extranuclear signalling by steroid receptors. Nat. Rev. Mol. Cell Biol. 17, 783–797. 10.1038/nrm.2016.122 27729652 PMC5649368

[B31] Luine V. N. (2014). Estradiol and cognitive function: past, present and future. Horm. Behav. 66, 602–618. 10.1016/j.yhbeh.2014.08.011 25205317 PMC4318702

[B32] Luine V. N. Frankfurt M. (2012). Estrogens facilitate memory processing through membrane mediated mechanisms and alterations in spine density. Front. Neuroendocrinol. 33, 388–402. 10.1016/j.yfrne.2012.07.004 22981654 PMC3496031

[B33] MacLusky N. J. Luine V. N. Hajszan T. Leranth C. (2005). The 17alpha and 17beta isomers of estradiol both induce rapid spine synapse formation in the CA1 hippocampal subfield of ovariectomized female rats. Endocrinology 146, 287–293. 10.1210/en.2004-0730 15486220

[B34] MacQueen G. M. Campbell S. McEwen B. S. Macdonald K. Amano S.. (2003). Course of illness, hippocampal function, and hippocampal volume in major depression. Proc. Natl. Acad. Sci. U.S.A. 100, 1387–1392. 10.1073/pnas.0337481100 12552118 PMC298782

[B35] Mannella P. Brinton R. D. (2006). Estrogen receptor protein interaction with phosphatidylinositol 3-kinase leads to activation of phosphorylated Akt and extracellular signal-regulated kinase 1/2 in the same population of cortical neurons: a unified mechanism of estrogen action. J. Neurosci. 26, 9439–9447. 10.1523/JNEUROSCI.1443-06.2006 16971528 PMC6674594

[B36] Migliaccio A. Castoria G. Di Domenico M. de Falco A. Bilancio A.. (2000). Steroid-induced androgen receptor-oestradiol receptor beta-Src complex triggers prostate cancer cell proliferation. EMBO J. 19, 5406–5417. 10.1093/emboj/19.20.5406 11032808 PMC314017

[B37] Migliaccio A. Di Domenico M. Castoria G. de Falco A. Bontempo P.. (1996). Tyrosine kinase/p21ras/MAP-kinase pathway activation by estradiol-receptor complex in MCF-7 cells. EMBO J. 15, 1292–1300. 8635462 PMC450032

[B38] Milner T. A. Ayoola K. Drake C. T. Herrick S. P. Tabori N. E.. (2005). Ultrastructural localization of estrogen receptor beta immunoreactivity in the rat hippocampal formation. J. Comp. Neurol. 491, 81–95. 10.1002/cne.20724 16127691

[B39] Mukai H. Hatanaka Y. Mitsuhashi K. Hojo Y. Komatsuzaki Y.. (2011). Automated analysis of spines from confocal laser microscopy images: application to the discrimination of androgen and estrogen effects on spinogenesis. Cereb Cortex. 21, 2704–2711. 10.1093/cercor/bhr059 21527787 PMC3209797

[B40] Mukai H. Kimoto T. Hojo Y. Kawato S. Murakami G.. (2010). Modulation of synaptic plasticity by brain estrogen in the hippocampus. Biochim. Biophys. Acta 1800, 1030–1044. 10.1016/j.bbagen.2009.11.002 19909788

[B41] Mukai H. Tsurugizawa T. Murakami G. Kominami S. Ishii H.. (2007). Rapid modulation of long-term depression and spinogenesis via synaptic estrogen receptors in hippocampal principal neurons. J. Neurochem. 100, 950–967. 10.1111/j.1471-4159.2006.04264.x 17266735

[B42] Murakami G. Hojo Y. Ogiue-Ikeda M. Mukai H. Chambon P.. (2015). Estrogen receptor KO mice study on rapid modulation of spines and long-term depression in the hippocampus. Brain Res. 1621, 133–146. 10.1016/j.brainres.2014.12.002 25498865

[B43] Murakami G. Tsurugizawa T. Hatanaka Y. Komatsuzaki Y. Tanabe N.. (2006). Comparison between basal and apical dendritic spines in estrogen-induced rapid spinogenesis of CA1 principal neurons in the adult hippocampus. Biochem. Biophys. Res. Commun. 351, 553–558. 10.1016/j.bbrc.2006.10.066 17070772

[B44] Nguyen T. V. Yao M. Pike C. J. (2005). Androgens activate mitogen-activated protein kinase signaling: role in neuroprotection. J. Neurochem. 94, 1639–1651. 10.1111/j.1471-4159.2005.03318.x 16011741

[B45] Okamoto M. Hojo Y. Inoue K. Matsui T. Kawato S.. (2012). Mild exercise increases dihydrotestosterone in hippocampus providing evidence for androgenic mediation of neurogenesis. Proc. Natl. Acad. Sci. U.S.A. 109, 13100–13105. 10.1073/pnas.1210023109 22807478 PMC3420174

[B46] Ooishi Y. Kawato S. Hojo Y. Hatanaka Y. Higo S.. (2012a). Modulation of synaptic plasticity in the hippocampus by hippocampus-derived estrogen and androgen. J Steroid Biochem Mol. Biol. 131, 37–51. 10.1016/j.jsbmb.2011.10.004 22075082

[B47] Ooishi Y. Mukai H. Hojo Y. Murakami G. Hasegawa Y.. (2012b). Estradiol rapidly rescues synaptic transmission from corticosterone-induced suppression via synaptic/extranuclear steroid receptors in the hippocampus. Cereb Cortex 22, 926–936. 10.1093/cercor/bhr164 21725036

[B48] Pedram A. Razandi M. Levin E. R. (2006). Nature of functional estrogen receptors at the plasma membrane. Mol. Endocrinol. 20, 1996–2009. 10.1210/me.2005-0525 16645038

[B49] Pedram A. Razandi M. Sainson R. C. Kim J. K. Hughes C. C. Levin E. R. (2007). A conserved mechanism for steroid receptor translocation to the plasma membrane. J. Biol. Chem. 282:22278. 10.1074/jbc.M611877200 17535799

[B50] Phan A. Gabor C. S. Favaro K. J. Kaschack S. Armstrong J. N.. (2012). Low doses of 17beta-estradiol rapidly improve learning and increase hippocampal dendritic spines. Neuropsychopharmacology 37, 2299–2309. 10.1038/npp.2012.82 22669167 PMC3422494

[B51] Phan A. Lancaster K. E. Armstrong J. N. MacLusky N. J. Choleris E. (2011). Rapid effects of estrogen receptor alpha and beta selective agonists on learning and dendritic spines in female mice. Endocrinology 152, 1492–1502. 10.1210/en.2010-1273 21285321

[B52] Rusanescu G. Qi H. Thomas S. M. Brugge J. S. Halegoua S. (1995). Calcium influx induces neurite growth through a Src-Ras signaling cassette. Neuron 15, 1415–1425. 10.1016/0896-6273(95)90019-5 8845164

[B53] Shinohara Y. Hirase H. Watanabe M. Itakura M. Takahashi M. Shigemoto R. (2008). Left-right asymmetry of the hippocampal synapses with differential subunit allocation of glutamate receptors. Proc. Natl. Acad. Sci. U.S.A. 105, 19498–19503. 10.1073/pnas.0807461105 19052236 PMC2593619

[B54] Simerly R. B. Chang C. Muramatsu M. Swanson L. W. (1990). Distribution of androgen and estrogen receptor mRNA-containing cells in the rat brain: an *in situ* hybridization study. J. Comp. Neurol. 294, 76–95. 10.1002/cne.902940107 2324335

[B55] Tabori N. E. Stewart L. S. Znamensky V. Romeo R. D. Alves S. E.. (2005). Ultrastructural evidence that androgen receptors are located at extranuclear sites in the rat hippocampal formation. Neuroscience 130, 151–163. 10.1016/j.neuroscience.2004.08.048 15561432

[B56] Tuscher J. J. Luine V. Frankfurt M. Frick K. M. (2016). Estradiol-Mediated spine changes in the dorsal hippocampus and medial prefrontal cortex of ovariectomized female mice depend on ERK and mTOR activation in the dorsal hippocampus. J. Neurosci. 36, 1483–1489. 10.1523/JNEUROSCI.3135-15.2016 26843632 PMC4737764

[B57] Weed S. A. Du Y. Parsons J. T. (1998). Translocation of cortactin to the cell periphery is mediated by the small GTPase Rac1. J. Cell Sci. 111 (Pt 16), 2433–2443. 9683637 10.1242/jcs.111.16.2433

[B58] Woolley C. S. Gould E. McEwen B. S. (1990). Exposure to excess glucocorticoids alters dendritic morphology of adult hippocampal pyramidal neurons. Brain Res. 531, 225–231. 10.1016/0006-8993(90)90778-A 1705153

[B59] Woolley C. S. McEwen B. S. (1992). Estradiol mediates fluctuation in hippocampal synapse density during the estrous cycle in the adult rat. J. Neurosci. 12, 2549–2554. 1613547 10.1523/JNEUROSCI.12-07-02549.1992 PMC6575846

[B60] Znamensky V. Akama K. T. McEwen B. S. Milner T. A. (2003). Estrogen levels regulate the subcellular distribution of phosphorylated Akt in hippocampal CA1 dendrites. J. Neurosci. 23, 2340–2347. 10.1523/JNEUROSCI.23-06-02340.2003 12657693 PMC6742003

